# Circular RNA S-7 promotes ovarian cancer EMT via sponging miR-641 to up-regulate ZEB1 and *MDM2*

**DOI:** 10.1042/BSR20200825

**Published:** 2020-07-24

**Authors:** Fenghua Zhang, Yun Xu, Wenfeng Ye, Jingting Jiang, Changping Wu

**Affiliations:** 1 Department of Obstetrics and Gynecology, the Third Affiliated Hospital of Soochow University, 185 Bureau Street, Changzhou 213003, China; 2 Changzhou Cancer Biomedical Treatment Center of Jiangsu Province, 185 Bureau Street, Changzhou 213003, China; 3 Department of Oncology, the Third Affiliated Hospital of Soochow University, 185 Bureau Street, Changzhou 213003, China

**Keywords:** biomarkers, non-coding RNA, ovarian cancer

## Abstract

Background: Ovarian cancer (OC) is one lethal gynecologic cancer, with a 5-year survival rate approximately 47% and localized stage diagnosis of 15%. Circular RNAs are promising biomarkers for malignancies.

Methods: CiRS-7 expression was confirmed in 40 paired OC and normal adjacent tissues from 40 OC patients with different TNM stages, lymph node metastasis status and overall survival rate, also 5 different OC cell lines by qRT-PCR. Effects of ciRS-7 silence on OC cell phenotypes were determined in OC cells and Xenograft mouse model. StarBase was used to predict binding sites between ciRS-7 and micRNAs. Pearson correlation analysis and RNA-immunoprecipitation assay were used to determine the association between genes. Point mutation and rescue experiments were applied for molecular mechanism investigation.

Results: CiRS-7 expression was significantly higher in OC cells and tissues, which was significantly associated with the TNM stages, lymph node metastasis status and overall survival rate in OC patients. CiRS-7 silence inhibited OC cell growth and metastasis. CiRS-7 sponged miR-641 to up-regulate ZEB1 and MDM2 expression in OC development.

Conclusion: CiRS-7 serves as a competing endogenous RNA of miR-641 that promoted cell growth and metastasis in OC, via regulating ZEB1 and MDM2-mediated EMT. High ciRS-7 expression was a poor prognosis of TNM stages, lymph node metastasis status and overall survival rate in OC patients. Targeting ciRS-7/miR-641/ZEB1 or ciRS-7/miR-641/MDM2 axis may be a novel diagnostic, prognostic and therapeutic strategy for OC.

## Background

Ovarian cancer (OC) is still the seventh most regular fatal gynecological malignancy, leading to a predictable 125,000 global deaths each year [1]. Though platinum-based regimen has been applied beyond four decades, only slight improvement has been observed in the survival and prognosis of OC patients [2,3]. Approximately 90% of all OC are epithelial malignancy, which accounts for the massive majority of the OC-associated deaths [3]. Therefore, many efforts have been devoted to the investigation and management of epithelial OC.

It is well known that the epithelial–mesenchymal transition (EMT) plays an essential role in cancer metastasis, since gaining invasive and metastatic ability is achieved by the epithelial characteristics loss [4,5]. Identifying factors that inhibit EMT should, thus, assist the improvement of effective clinical managements.

Non-coding RNAs (ncRNAs) including microRNA (miRNA) and long non-coding RNA (lncRNA) have been found to play essential roles in tumorigenesis [6,7].

Circular RNAs (circRNAs) are a novel cluster of ncRNAs with basically unclear biological functions. Evidences confirmed that circRNAs are rich in cells showing cell/tissue-specific expression [8]. With a covalently closed continuous loop structure, crcRNAs regulate gene expression through competitive binding to miRNAs [9–11], thus to serve as miRNA sponges and produce the circRNA–miRNA–mRNA axis [12–18].

CircRNAs also are potential biomarkers for many disease diagnosis such as cancer [19]. As a result, functions of circRNAs in the carcinogenesis have recently fascinated researcher's attention. The circRNA regulated gene expression varies in different categories or different stages of cancers [8]. Abnormal expression of circRNAs during OC progress has been previously reported [20]. Therefore, it is important to identify the dysregulated circRNAs, explore novel molecular mechanisms and therapeutic targets for OC management.

Recently, a novel circRNA, ciRS-7 was reported to be a potential prognostic biomarker in colorectal cancer patients [21]. However, its function and clinical relevance in OC are unknown.

In the present study, we aimed to explore the function, molecular mechanisms and clinical implications of the ciRS-7 in OC, using the patent tumor tissues, cells and animal models for a comprehensive understanding and solid evidences. Our results showed that ciRS-7 was up-regulated in OC cells and tissues, in turn, promoting cell proliferation and the EMT. More importantly, we found that ciRS-7 functioned as a sponge of miR-641 to up-regulate the ZEB1 and MDM2 expression and consequently promoted the tumorigenesis of OC. The present study provided novel evidences that may help the development of effective therapeutic strategies against OC in the clinical practices.

## Methods

### Patient tissues

The OC and paired adjacent normal ovarian tissues were obtained from 40 OC patients experiencing surgery at the department of obstetrics and gynecology, the third affiliated hospital of soochow university. All specimens were snap frozen in liquid nitrogen at the time of collection and stored at −80°C until use.

### Reagents

Macoy's 5A medium and fetal bovine serum (FBS) were from Gibco (Rockford, MD, U.S.A.). PrimeScrip™ RT Master Mix, RNAiso Plus and SYBR Green Premix Ex Taq™ II were from TaKaRa (Dalian, China). MiRNeasy Mini Kit, miScript II RT Kit and miScript SYBR Green PCR Kit were from Qiagen (Duesseldorf, Germany). Matrigel was from BD (New Jersey, U.S.A.). CCK-8 assay kit was from Dojindo Corp (Kyushu, Japan). SuperSignal West Dura Extended Duration Substrate was from Thermo Fisher (IL, U.S.A.). RIPA lysis buffer was from Beyotime (Shanghai, China). Antibodies against ZEB1, MDM2 and GAPDH were bought from Santa Cruz Biotechnology, Inc. (Dallas, U.S.A.).

### Cell lines and cell culture

Five OC cell lines (SKOV3, A2780, OV2008, IGROV1 and ES-2) and a normal human ovarian epithelial cell line (HOSE) were used. SKOV3 and ES-2 cells were bought from American Type Culture Collection (Manassas, VA, U.S.A.). A2780, OV2008, IGROV1 and HOSE cells were from Shanghai Baiyi Biotechnology Center. The cells were cultured in Macoy’s 5A medium containing FBS (10%), penicillin (100 IU/ml) and streptomycin (100 mg/ml) at 37°C and a humidified atmosphere with 5% CO_2_.

### Target gene prediction

The potential sponged miRNAs by the ciRS-7 were predicted via the website tool starBase (http://starbase.sysu.edu.cn). The target mRNAs regulated by miR-641 were determined based on the literatures.

### Quantitative real-time PCR (qRT-PCR)

The purity and concentration of total RNA isolated with the RNAiso Plus were evaluated using the NanoDrop ND-1000 (Thermo Fisher Scientific, Wilmington, DE). PrimeScrip™ RT Master Mix was used for reverse transcription, and SYBR Green Premix Ex Taq™ II was used for cDNA amplification following the manufacturer’s instructions. Following the manufacturer’s instructions, a miRNeasy Mini Kit was applied to isolate miRNAs, a miScript II RT Kit was used for reverse transcription and a miScript SYBR Green PCR Kit was used for cDNA amplification. A qRT-PCR was performed on an AB7300 thermo recycler (Applied Biosystems, Carlsbad, CA) using the TaqMan Universal PCR Master Mix. Relative gene expression was calculated by 2^−ΔΔCt^ method using GAPDH as the internal control for mRNA and circRNA, and U6 as the internal control for miRNA.

The primers are as follows:

ciRS-7 (forward-5′-ACGTCTCCAGTGTGCTGA-3′, reverse -5′-CTTGACACAGGTGCCATC-3′);
miR-641 (forward-5′-GGGGAAAGACATAGGATAGAGT-3′, reverse-5′- CAGTGCGTGTCGTGGAG-3′);
*zeb1* (forward- 5′-GATGATGAATGCGAGTCAGATGC-3′, reverse- 5′-ACAGCAGTGTCTTGTTGTTGT-3′);
*mdm2* (forward- 5′-CAGTAGCAGTGAATCTACAGGGA-3 ′, reverse- 5′-CTGATCCAACCAATCACCTGAAT-3 ′);
*u6* (forward-5′- TOCTTCGOCAOCACATATAC-3 ′, reverse-5′- AGGGOCCATOCTAATCTTCT-3′);
*gapdh* (forward-5′-TOCACCACCAACTOCTTAOC-3′, reverse-5 ′-GOCATGGACTGTGGTCATGAG-3′).

### Cell proliferation assay

CCK-8 kit was used for the cell proliferation assay following the manufacturer’s instructions. In brief, 10 μl of CCK-8 reagent was directly added into the culture medium of the cells to be tested, which were cultured in a 96-well plate (3000 cells/well) for the indicated time. After incubation for 2.5 h at 37°C, the optional density (OD) at 450 nm was measured.

### Colony formation assay

Cells were seeded in a six-well plate (1 × 10^3^ cells/well) and cultured for 7 days at 37°C with fresh medium supplementation every two days. On theseventh day, cells were fixed in 4% paraformaldehyde and stained with a Crystal Violet solution. Cell colonies were then counted, and the pictures were obtained.

### Migration and invasion assay

The 24-well transwells (Corning Costar, 8.0 μm pore size) were used for the cell migration and invasion assay. For the migration assay, the transwell filter without coated matrigel on the upper surface was used; while for the invasion assay, the transwell filter coated with matrigel on the upper surface was used. In brief, 3 × 10^5^ cells in 200 μl of serum-free medium were seeded on the upper chamber, 500 μl of medium containing 10% FBS was loaded into the lower chamber. After 48-h incubation, the non-migrated and non-invaded cells on the upper surface of the chamber were scraped off using a cotton swab, and the cells migrated or invaded to the opposite side were fixed in 4% paraformaldehyde and stained with a crystal violet solution. The cell numbers were then counted and compared.

### Xenograft nude mouse model establishment and analysis

Four-week-old male BALB/c nude mice, weighing 18–21 g, were bought from Shanghai SLAC Laboratory Animal Co, Ltd. (Shanghai, China), and housed in a temperature-controlled and pathogen-free environment at the Animal Experiment Center of soochow University. All processes and animal experiments used in this study were approved by the Animal Care and Use Committee of soochow university and performed at the Animal Experiment Center of Soochow University.

About 5 × 10^6^ SKOV3 cells in PBS (200 ml) were injected into the right flanks of each mouse. About one week after the injection, when the subcutaneous tumor volume was about 100 mm^3^, the mice were randomly divided into a si-NC group (*n*=6), a si-ciRS-7 group (*n*=6), a si-ciRS-7+inh-miR-641 group (*n*=6), then the cholesterol-modified si-NC, si-ciRS-7, si-ciRS-7+inh-miR-641 oligonucleotide was injected into the tumors of the designated group respectively, with two times a week for 2 weeks. The long diameter (*a*) and short diameter (*b*) of each orthotopic tumor were measured with a caliper every 3 days to calculate the tumor volume (*V*): *V* = 1/2 × *a* × *b*^2^, then the tumor growth curves were plotted. Mice were killed under the euthanasia by mask inhalation of isoflurane vaporized at concentrations of 1.5%, 21 days after the injection, the tumor tissues were isolated and weighed.

### Western blotting analysis

Total proteins from OC cells were isolated with the RIPA lysis buffer. Equal amounts of proteins were separated on a 10% SDS-PAGE gel, transferred onto the PVDF membranes and incubated with the diluted primary antibodies overnight at 4°C, followed by incubation with a HRP rabbit IgG secondary antibody at room temperature for 1 h. The membranes were washed three times with TBST and visualized using SuperSignal West Dura Extended Duration Substrate according to the manufacturer’s instructions.

### Statistical analysis

Statistical analyses were performed using SPSS 20.0 (IBM, SPSS, Chicago, IL, U.S.A.) and GraphPad Prism (GraphPad, La Jolla, U.S.A.). Student’s *t*-test or Chi-square test was used to estimate the statistical significance between groups. The association between genes was evaluated by Pearson correlation analysis. *P*<0.05 indicated statistically significant.

## Results

### High expression level of ciRS-7 indicated poor prognosis for TNM stages, lymph node metastasis status and overall survival rate in OC patients

**Figure 1:**
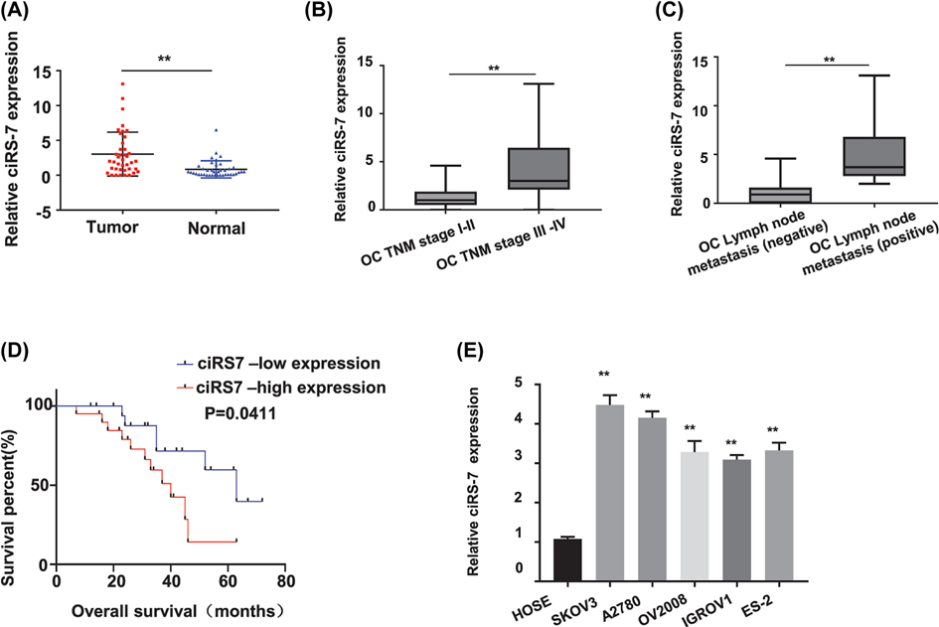
CiRS-7 was up-regulated in OC tissues and an independent prognostic factor for TNM stages, lymph node metastasis status and overall survival in OC patients (**A**) relative ciRS-7 expression was statistically significantly higher in OC tissues (*n*=40) than in paired normal adjacent tissues (*n*=40) from 40 OC patients detected by qRT-PCR. (**B**) Relative ciRS-7 expression in OC tissues from stage III-IV patients (*n*=21) was statistically significantly higher than that in the OC tissues from stage I-II patients (*n*=19) detected by qRT-PCR. (**C**) Relative ciRS-7 expression level was statistically significantly higher in OC tissues of the positive lymph node metastasis group (*n*=17) than that in the negative lymph node metastasis group (*n*=23). (**D**) Overall survival rates were statistically significantly decreased in OC patients with high ciRS-7 expression. (**E**) Relative ciRS-7 expression was statistically significantly higher in the OC cells (SKOV3, A2780, OV2008, IGROV1, ES-2) than in the normal human cervical epithelial HOSE cells. Relative ciRS-7 expression was calculated by 2^−ΔΔCt^ method using GAPDH as the internal control. All the experiments were repeated for three times; **P<0.01

To investigate the clinical association of ciRS-7 expression level in OC patients, 40 pairs of OC and self-matched adjacent normal tissues were collected from the OC patients. The ciRS-7 expression level in these 40 OC patients was first determined by qRT-PCR since it has not been reported. Our results showed that the ciRS-7 expression level was significantly increased in the OC tissues than that in the adjacent normal tissues (Figure 1A, *P*<0.01). To explore whether the high ciRS-7 expression level is correlated with the advanced tumor stage, the lymph node metastasis status or the overall survival rate in the OC patients, we next examined the expression patterns of ciRS-7 in the 40 OC patients with different TNM stages, lymph node metastasis status and overall survival rate. Our results found that the ciRS-7 expression level was significantly higher in the stage III-IV OC patients (*n*=21) than that in the stage I-II OC patients (*n*=19) (*P*<0.01, Figure 1B), also significantly higher in the OC tissues with lymph node metastasis (*n*=17) than those without the lymph node metastasis (*n*=23) (*P*<0.01, Figure 1C); patients with high ciRS-7 expression level showed a decreased overall survival rate (*P*=0.0411, Figure 1D). We further confirmed the ciRS-7 expression status in OC by comparing the ciRS-7 expression levels between the OC cells and the normal human ovarian epithelial cells. As shown in Figure 1E, ciRS-7 expression level was dramatically higher in all tested OC cells (SKOV3, A2780, OV2008, IGROV1 and ES-2) than that in the normal human ovarian epithelial HOSE cells (*P*<0.01). All these data indicated that the up-regulated ciRS-7 expression level was significantly related to the poor prognosis of OC patients with advanced TNM stages, lymph node metastasis, and the decreased overall survival rate.

### CiRS-7 induced OC growth both *in vitro* and *in vivo*

**Figure 2:**
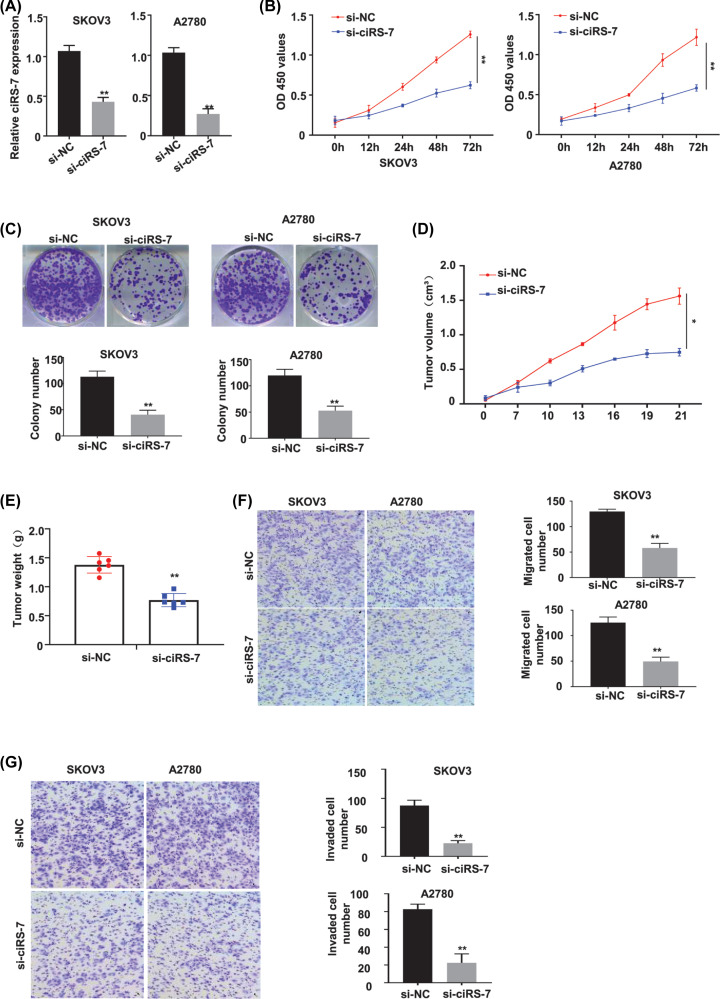
CiRS-7 silence inhibited proliferation, migration and invasion of OC cells *in vitro* as well as tumor growth *in vivo* (**A**) qRT-PCR analysis confirmed that the relative ciRS-7 expression, calculated by 2^−ΔΔCt^ method using GAPDH as the internal control, was successfully silenced by si-ciRS-7 in the SKOV3 and the A2780 cells, the following phenotypes were then evaluated in the SKOV3 and A2780 cells transfected with the si-NC or si-ciRS-7: cell viability detected by CCK-8 assay *in vitro* (**B**) colony number detected by colony formation assay *in vitro* (**C**) tumor volume (**D**) and tumor weight (**E**) in the si-NC group (*n*=6) and the si-ciRS-7 group (*n*=6) detected *in vivo* using the xenograft mouse model; cell migration (**F**) and cell invasion (**G**) detected by transwell assay. All the experiments were repeated for three times; *P<0.01; **P<0.01.

To determine the biological roles of ciRS-7 in OC, two OC cell lines (SKOV3 and A2780) with the highest ciRS-7 expression level were selected to silence the ciRS-7 expression by transfection of the siRNA targeting ciRS-7 (designated as si-ciRS-7). We found that si-ciRS-7 transfection resulted in a statistically significant decrease in the ciRS-7 expression level of both the SKOV3 and A2780 cells (*P*<0.01, Figure 2A); compared with the negative control (si-NC), si-ciRS-7 transfection knocked down more than 50% of ciRS-7 expression in both SKOV3 and A2780 cells detected by qRT-PCR assay (Figure 2A). Furthermore, the CCK-8 assay was used to detect the cell viability of SKOV3 and A2780 cells after transfection of si-NC or si-ciRS-7 for 0, 12, 24, 48 and 72 h, our results showed that ciRS-7 silence statistically significantly inhibited the viability (OD value) of both SKOV3 (Figure 2B, left panel) and A2780 (Figure 2B, right panel) cells at 450 nm (*P*<0.01); colony formation assay indicated that ciRS-7 silence statistically significantly inhibited the colony formation ability (*P*<0.01), the images (Figure 2C, the upper panels) and numbers (Figure 2C, the lower panels) of the colonies were acquired under the microscope; meanwhile, silence of ciRS-7 decreased the tumor volume (*P*<0.05, Figure 2D) and tumor weight (*P*<0.01, Figure 2E) of the OC xenografts in the nude mice. No dead mice and side effects were reported. All these data indicated that ciRS-7 promoted the proliferation of OC cells both *in vitro* and *in vivo.*

### CiRS-7 promoted EMT of OC cells

We all know that EMT plays an important role in promoting the malignant cell metastasis. So, we applied the transwell assays for the migration and the invasiveness to determine whether the EMT phenotypes of the CG cells were amplified by ciRS-7. As we can see, the migration (Figure 2F) and invasion (Figure 2G) capabilities of the OC cells were dramatically repressed with ciRS-7 knockdown (*P*<0.01), the photographs (Figure 2F,G, left panels), the migrated (Figure 2F, right panels) and invaded (Figure 2G, right panels) cell numbers were attained by a microscope. These data indicated that ciRS-7 stimulated the metastasis of OC cells.

### CiRS-7 was confirmed to sponge miR-641 in OC cells

**Figure 3:**
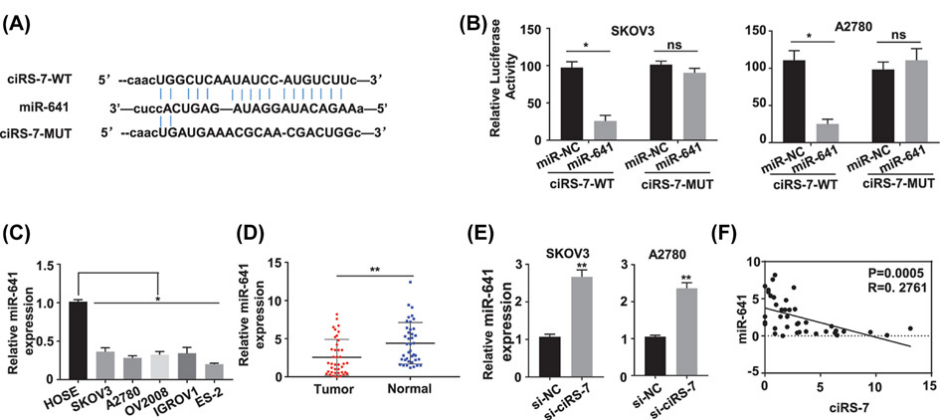
Identification of the potential miRNA sponged by ciRS-7 (**A**) Schematic representation of the predicted potential binding sites of miR-641 with ciRS-7 (http://starbase.sysu.edu.cn), and the mutation sites for specific assay. (**B**) Double luciferase reporter assay in the SKOV3 and A2780 cells co-transfected with ciRS-7 WT or mutated reporter and miR-641 mimics. (**C**) The miR-641 expression level was down-regulated in the human OC cells (SKOV3, A2780, OV2008, IGROV1 and ES-2) than in the normal human cervical epithelial HOSE cells tested by qRT-PCR. (**D**) The miR-641 expression levels in paired OC (*n*=40) and adjacent normal (*n*=40) tissues from 40 OC patients (same samples as in Figure 1A) detected by qRT-PCR. (**E**) The miR-641 expression levels in ciRS-7 silenced SKOV3 and A2780 cells detected by qRT-PCR. (**F**) Pearson correlation analysis of the association between miR-641 with ciRS-7 in the OC tissues from 40 OC patients (same samples as in Figure 1A). Relative miR-641 expression was calculated by 2^−ΔΔCt^ method using U6 as the internal control. All the experiments were repeated for three times; *P<0.01; **P<0.01.

As previously revealed, circRNAs principally served as spongers of miRNAs to regulate the target gene expression. Hence, we investigated the possible miRNAs sponged by the ciRS-7. The starBase (http://starbase.sysu.edu.cn) was first applied to predict the target miRNAs which might to be bind by the ciRS-7 sequence, and miR-641 was selected as the most prospective target of ciRS-7(Figure 3A). To further explore if miR-641 communicated with ciRS-7 in OC cells, luciferase reporter gene assay was carried out in SKOV3 and A2780 cells, respectively, the data displayed that up-regulation of miR-641 repressed the luciferase activities of SKOV3 (*P*<0.05, Figure 3B, left panel) and A2780 (*P*<0.05, Figure 3B, right panel) cells with the wild-type ciRS-7 reporter gene, this efficacy was vanished once the expected ciRS-7-binding site with miR-641 was mutated (Figure 3B). Moreover, the functional relationship of ciRS-7 and miR-641 was explored by detecting miR-641 level in the OC tissues and cells, the data showed that miR-641 level was dramatically decreased in the OC cells (SKOV3, A2780, OV2008, IGROV1 and ES-2) versus the HOSE cells (*P*<0.05, Figure 3C); in OC tissues (*n*=40) versus matched nearby normal tissues (*n*=40) of 40 OC patients identified with qRT-PCR (*P*<0.01, Figure 3D); meanwhile, the miR-641 level in SKOV3 and A2780 cells with ciRS-7 silence was dramatically up-regulated (*P*<0.01, Figure 3E). The association between ciRS-7 with miR-641 was further investigated with Pearson correlation coefficient assay, the results showed that the ciRS-7 expression level was significantly negatively related to miR-641 expression level in the 40 OC patient tissues (*P*<0.001, Figure 3F). These results suggested that ciRS-7 straightly communicated with miR-641, leading to the metastasis and invasion of the OC cells.

### CiRS-7 sponged miR-641 to up-regulate zeb1 and mdm2 in OC cells

**Figure 4:**
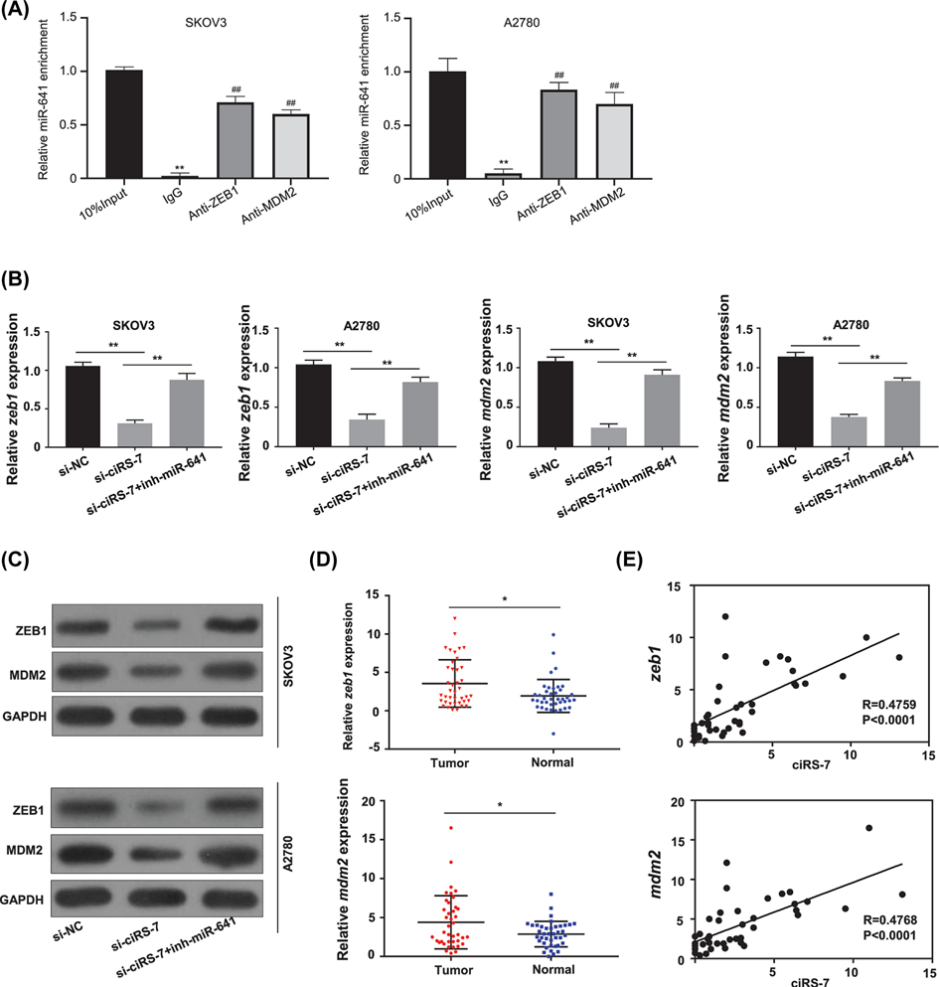
ciRS-7 increased the expression levels of oncogenic *zeb1* and *mdm2* by sponging miR-641 in OC development (**A**) The interactions between the miR614 and ZEB1, the miR-614 and MDM2 in both the SKOV3 (left panel) and A2780 (right panel) cells were detected by RNA-immunoprecipitation assay. (**B**) The mRNA expression levels of *zeb1* and *mdm2* in the SKOV3 and A2780 cells after ciRS-7 knockdown (si-ciRS-7) with or without miR-641 inhibitor (inh-miR-641) detected by qRT-PCR. (**C**) The protein expression levels of ZEB1 and MDM2 in the SKOV3 and A2780 cells after ciRS-7 knockdown with or without inh-miR-641 detected by Western blot. (**D**) The expression levels of *zeb1* and *mdm2* in paired OC (*n*=40) and adjacent normal tissues (*n*=40) from 40 patients (same samples as in Figure 1A) detected by qRT-PCR. (**E**) Pearson correlation analysis of the association between *zeb1* or *mdm2* with ciRS-7 in the OC tissues (*n*=40) from 40 OC patients (same samples as in Figure 1A). Relative *zeb1* and *mdm2* expression levels were calculated by 2^−ΔΔCt^ method using GAPDH as the internal control. All the experiments were repeated for three times; *P<0.01; **P<0.01

It has been reported that *zeb1* and *mdm2* are the downstream targets of miR-641 [22,23]; meanwhile, RNA-immunoprecipitation assay showed the direct interaction between the miR-614 and ZEB1, the miR-614 and MDM2 in both the SKOV3 and A2780 cells (Figure 4A). To determine whether ciRS-7 sponges miR-641 to regulate *zeb1* and *mdm2* expression, we first determined the *zeb1* and *mdm2* expression levels by qRT-PCR in the OC cells. Our results found that ciRS-7 silence considerably down-regulated the mRNA expression levels of *zeb1* and *mdm2* (*P*<0.01, Figure 4B) and also the protein expression levels of ZEB1 and MDM2 (Figure 4C) in both the SKOV3 and A2780 cells, while the findings were somewhat reversed after cotransfecting the inhibitor for miR-641 (inh-miR-641) (Figure 4B,C). The *zeb1* and *mdm2* expression levels of the 40 OC patient tissues were measured by qRT-PCR, the results showed that both *zeb1* and *mdm2* levels were significantly amplified in the OC tissues versus the nearby normal tissues (Figure 4D, *P*<0.05). In the meantime, the relationships between ciRS-7 and *zeb1* or *mdm2* expression levels in the 40 OC patient tissues were evaluated with the Pearson correlation assay, and the results showed that that ciRS-7 expression level was positively and dramatically related with *zeb1* and *mdm2* expression levels (Figure 4E, *P*<0.0001).

**Figure 5:**
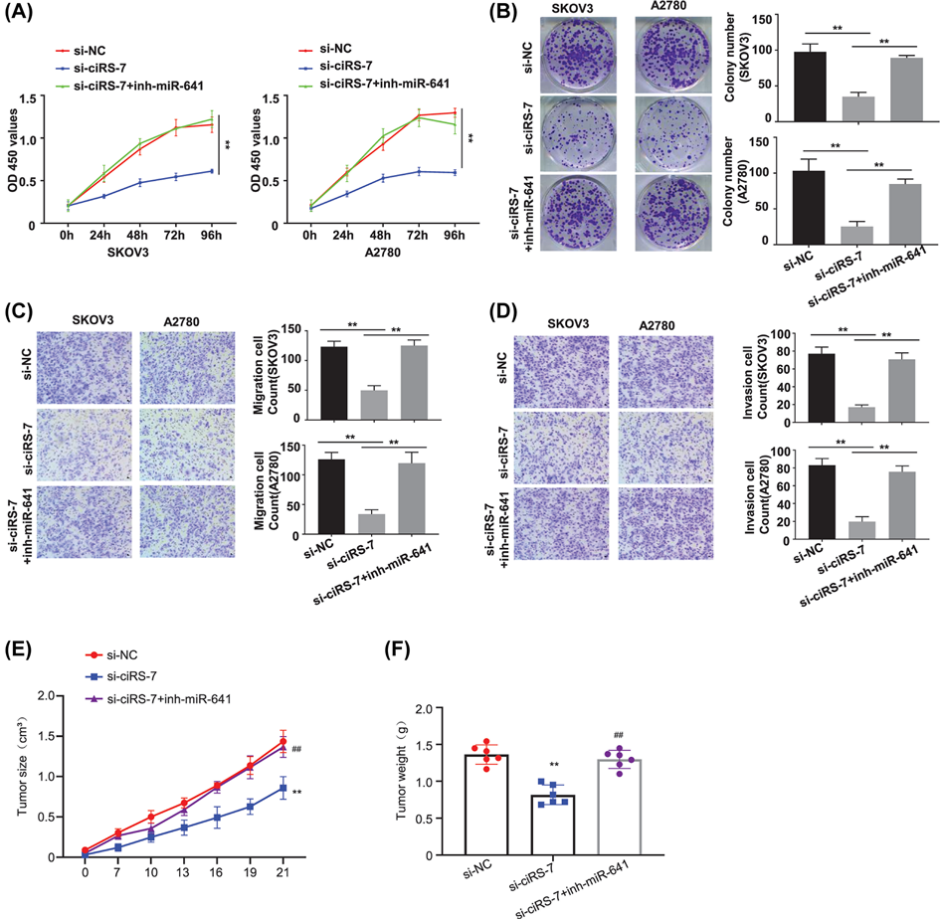
Ablation of miR-641 rescued the decreased malignant phenotype induced by ciRS-7 silence in OC cells (**A**) Cell viability in SKOV3 and A2780 cells after ciRS-7 knockdown with or without inh-miR-641 detected by CCK-8 assay. (**B**) Colony formation assay in SKOV3 and A2780 cells after ciRS-7 knockdown with or without inh-miR-641. (**C**) Cell migration ability in SKOV3 and A2780 cells after ciRS-7 knockdown with or without inh-miR-641 detected by the transwell assay without matrigel. (**D**) Cell invasion ability in SKOV3 and A2780 cells after ciRS-7 knockdown with or without inh-miR-641 detected by the transwell assay with matrigel; tumor volume (**E**) and tumor weight (**F**) in the si-NC group (*n*=6), and the si-ciRS-7 without inh-miR-641(si-ciRS-7) group (*n*=6) and the si-ciRS-7 with inh-miR-641 group (si-ciRS-7+inh-miR-641) group (*n*=6) detected *in vivo* using the xenograft mouse model. All the experiments were repeated for three times; *P<0.01; **P<0.01; ^##^*P*<0.01.

Cell viability in SKOV3 and A2780 cells (transfected with si-NC, si-ciRS-7, si-ciRS-7+inh-miR-641) at different time points were detected by CCK-8 assay, our results showed that knockdown of ciRS-7 decreased the OD value at 450 nm, and this effect was partially reversed after co-transfection of the inh-miR-641 (Figure 5A, *P*<0.01).

The colony formation ability of the SKOV3 and A2780 cells (transfected with si-NC, si-ciRS-7, si-ciRS-7+inh-miR-641) was determined by the colony formation assay, which showed that ciRS-7 silence decreased the colony formation ability, and this effect was partially reversed by co-infection of the miR-641 inhibitor (Figure 5B, *P*<0.01).

Cell migration and invasion abilities of the SKOV3 and A2780 cells (transfected with si-NC, si-ciRS-7, si-ciRS-7+inh-miR-641) were investigated by the Transwell assay without or with the matrigel, our results showed that ciRS-7 silence reduced both the migration (Figure 5C, *P*<0.01) and invasion (Figure 5D, *P*<0.01) abilities, and this effect was partially reversed by co-transfection of the miR-641 inhibitor. A Tumor xenograft tumor model was then made to further confirm on tumor growth after subcutaneous inoculation with ciRS-7 knockdown SKOV3 cells with or without transfected miR-641 inhibitor. We demonstrated that the tumor proliferative activity, including the tumor volume (Figure 5E, *P*<0.01) and tumor weight (Figure 5F, *P*<0.01), was decreased in ciRS-7 down-regulated versus the control, and this effect was partially reversed by co-transfection of the miR-641 inhibitor.

## Discussion

Circular RNAs (circRNAs), which represent a new type of endogenous non-coding RNAs, are developed as frontiers in cancer research. To date, widespread expression of ciRS-7 in neuroblastomas, colorectal cancer, astrocytoma, renal cell, gastric cancer and pancreatic cancer [14,24,25] implicates the important character of ciRS-7 as a cancer regulator.

CiRS-7 is a recently recognized circRNA. In the present study, we investigated the OC and paired adjacent normal tissues from 40 OC patients with different clinical stages, lymph node metastasis status and overall survival rate. Here, we first revealed a significantly higher ciRS-7 expression in the OC tissues versus the matched adjacent normal tissues, suggesting its prospective oncogenic function in OC. Second, our data showed that high expression of ciRS-7 was significantly associated with the advanced clinical stages and lymph node metastasis status, as well as the poor overall survival rate of the OC patients, resulting in poor patient outcomes. This highlighted the applicability of ciRS-7 as a hopeful prognostic biomarker in OC patients. Third, from a biological perspective, we demonstrated that up-regulated ciRS-7 interrupted the normal function of miR-641 and thus stimulated the growth and EMT of OC cells. Fourth, we revealed a new mechanism that ciRS-7 served as a competing endogenous RNAs (ceRNA) and regulated ZEB1 and DM2 by competing the miR-641. Considering the critical role of ZEB1 and MDM2 signaling in OC development, our results for the first time discovered the therapeutic importance of ciRS-7 in OC patients.

MiRNA, a key component of the ncRNA family with a length of less than 22 nucleotides, plays complicated roles in governing the cellular functions by degradation of the target genes, thus to facilitate the cell growth, apoptosis, signaling transduction and oxidative response.

We used the on line tool starBase to predict the possible miRNA binding to ciRS-7, and miR-641 was found to be the best potential candidate. MiR-641 has been reported as a tumor suppressor in various cancers, such as the lung cancer [22] and cervical cancer [26]. In present study, we also found that miR-641 was down-regulated in both the OC cell lines and tissues, and served as an oncosuppressor in OC patents. MiR-641 expression level was significantly up-regulated when ciRS-7 was silenced in SKOV3 and A2780 cells; overexpression of miR-641 reduced the luciferase activities of the wild-type ciRS-7 reporter gene in SKOV3 and A2780 cells, while this repressed luciferase activity of ciRS-7 reporter gene was vanished when the predicted binding site of ciRS-7 with miR-641 was mutated; Pearson correlation analysis showed a negative association between the ciRS-7 and the miR-641 expression, these results suggested that ciRS-7 may act as a sponge to miR-641 in OC cells, ciRS-7 directly interacted with miR-641 to enable the metastasis and invasion of the OC cells.

The human proto-oncogene zinc finger E-box binding homeobox 1 (*zeb1*) is a transcription factor and plays essential role in regulating the normal physiological roles, such as the cell fate decisions, offering the cell stemness properties and cell plasticity [27,28]. High ZEB1 expression has been recognized in various human malignances, for example the lung cancer and hepatocellular carcinoma. ZEB1 regulates the EMT development, which accounts for the partial or whole transition of cancer cells with epithelial features to a mesenchymal status [29,30]. ZEB1 stimulates the migration and invasion of cancer cells through the EMT transition [31,32]. Therefore, ZEB1 is an encouraging prospective therapeutic target, ZEB1 inhibition may decrease the proliferation or metastasis of cancer cells.

HDM2 is the human homolog of the mouse/murine double minute 2 (*mdm2*) gene coded MDM2 protein and is an independent oncogene. These intrinsic characteristics make MDM2 a hopeful anti-cancer target and a molecular-based cancer biomarker [33].

It has been reported that miR-641 can target MDM2 in lung cancer and serve as a tumor suppressor; miRNA-641 inhibits the growth, migration and invasiveness, prompts cervical cancer cell apoptosis via straightly targeting *zeb1* [22,23]. We additional identified the considerably amplified mRNA levels of *zeb1* and *mdm2*. Pearson correlation analysis showed that ciRS-7 was positively and dramatically associated with *zeb1* and *mdm2* levels in OC tissues; ciRS-7 silence considerably down-regulated the mRNA and protein levels of both ZEB1 and MDM2, reduced the proliferation, colony formation, migration and invasive capabilities in OC cells, these properties were somewhat reversed by co-transfecting the inhibitor of miR-641, suggesting ZEB1 and MDM2 directly communicated with miR-641 via sponging ciRS-7 to encourage OC development. Consequently, ciRS-7 appeared to function as a ceRNA for miR-641 that promoted OC cell growth and metastasis through regulation of the ZEB1 or MDM2 mediated EMT. The effect of ciRS-7/miR-641/ZEB1 and ciRS-7/miR-641/MDM2 signaling on the metastasis of OC need to be further investigated using the mouse xenografts in the future.

## Conclusions

Our findings revealed that ciRS-7 expression level was significantly increased in OC patients and cells. High ciRS-7 expression level was significantly associated with a poor prognosis for TNM stages, lymph node metastasis and overall survival rate in OC patients. Functionally, ciRS-7 promoted OC cell growth, colony formation, migration and invasion by sponging miR-641 to up-regulate *zeb1* and *mdm2* expression, thus contributed to the OC cell EMT. Therefore, our data highlight the potential role of ciRS-7 as a novel oncogenic non-coding RNA that promotes the development of OC, dual targeting ciRS-7 and miR-641 may provide a novel therapeutic strategy to overcome this oncogenic pathway for OC patients.

## Data Availability

We declare to provide the data and materials in this study free of charge to the scientists for the non-commercial purposes.

## Abbreviations

ceRNA: competing endogenous RNA
circRNA: circular RNA
ciRS-7: circular RNA S-7
FBS: fetal bovine serum
miRNA: microRNA
ncRNA: non-coding RNA
OC: ovarian cancer
qRT-PCR: quantitative real-time PCR
