# Expression of Concern: Circular RNA S-7 promotes ovarian cancer EMT via sponging miR-641 to upregulate ZEB1 and MDM2

**DOI:** 10.1042/BSR-2020-0825_EOC

**Published:** 2024-04-29

**Keywords:** biomarkers, non-coding RNA, ovarian cancer

The Editorial Office has been made aware of potential issues surrounding the scientific validity of this paper, hence has issued an expression of concern to notify readers whilst the Editorial Office investigates. It has been noted that Figure 2C SKOV3 si-NC panel and A2780 si-NC panel seem to appear as Figure 5C SKOV3 sh-NC panel and OVCAR-3 sh-NC panel respectively from Cao et al, 2020 (DOI: 10.1111/jcmm.16185).

